# Decision makers perceptions and experiences of developing population-level interventions targeting risk factors for hypertension and diabetes in South Africa: a qualitative study

**DOI:** 10.1186/s12913-023-09135-x

**Published:** 2023-02-11

**Authors:** Lynn Hendricks, Jeannine Uwimana-Nicol, Taryn Young

**Affiliations:** 1 Division of Health Systems and Public Health, Department of Global Health, Faculty of Medicine and Health Sciences, Stellenbosch University, Cape Town, South Africa; 2 Social Research Methodology Group, Faculty of Social Sciences, KU Leuven, Leuven, Belgium; 3 School of Public Health, College of Medicine and Health Sciences, University of Rwanda, Kicukiro- Kigali, Rwanda; 4 Centre for Evidence Based Health Care, Division of Epidemiology and Biostatistics, Department of Global Health, Faculty of Medicine and Health Sciences, Stellenbosch University, Cape Town, South Africa

**Keywords:** Development, Planning, Monitoring and evaluation, Policy, Program, Risk factors, Diabetes, Hypertension, Qualitative, South Africa

## Abstract

**Background:**

People in low- and middle-income countries are disproportionately affected by Noncommunicable diseases (NCDs). NCD’s such as heart disease, cancer, chronic respiratory disease, and diabetes, are the leading cause of premature death worldwide and represent an emerging global health threat. The purpose of this qualitative study was to explore decision makers perceptions of developing population-level interventions (policies and programmes), targeting risk factors for hypertension and diabetes, in South Africa.

**Methods:**

Using purposive sampling we recruited fifteen participants, who were well informed about the policies, programs or supportive environment for prevention and management of diabetes and hypertension in South Africa. We conducted 12 individual interviews and 1 group interview (consisting of 3 participants). Data was analysed thematically in NVivo. The results were shared and discussed in two consultative stakeholder workshops, with participants, as part of a member validation process in qualitative research. All communication with participants was done virtually using MS Teams or ZOOM.

**Results:**

For development of population-level interventions, key enablers included, stakeholders’ engagement and collaboration, contextualization of policies and programs, and evaluation and organic growth. Challenges for supportive policy and program formulation, and to enable supportive environments, included the lack of time and resources, lack of consultation with stakeholders, regulations and competing priorities, and ineffective monitoring and evaluation. The main drivers of population-level interventions for diabetes and hypertension were perceived as the current contextual realities, costs, organizational reasons, and communication between various stakeholders.

**Conclusion:**

To address the risk factors for hypertension and diabetes in South Africa, policies and programs must account for the needs of the public and the historical and socio-economic climate. Feasibility and sustainability of programs can only be ensured when the resources are provided, and environments enabled to promote behavior change on a population-level. A holistic public health approach, which is contextually relevant, and evidence informed, is considered best practice in the formulation of population-level interventions.

**Supplementary Information:**

The online version contains supplementary material available at 10.1186/s12913-023-09135-x.

## Background

People in low- and middle-income countries (LMICs) are disproportionately affected by Noncommunicable diseases (NCDs). NCD’s such as heart disease, cancer, chronic respiratory disease, and diabetes, are the leading cause of premature death worldwide and represent an emerging global health threat, with approximately 41 million deaths each year (equivalent to 71% of deaths globally) [1]. Cardiovascular diseases (CVDs) account for more than 17.7 million deaths annually [2]. Hypertension, often asymptomatic, and diabetes are key risk factors for CVDs [3]. Out of all premature deaths due to NCDs more than 82% are in low- to upper-middle-income countries, and 37% are caused by CVDs [4, 5]. More than 80% of people living with diabetes (specifically type 2) are in LMICs [6, 7]. NCDs, instead of communicable diseases, are now demanding attention [8].

In line with the World Health Assembly’s call for a 25% reduction in NCD deaths by 2025 amongst the age group 30–70 years [9], the South African government committed to reduce the relative premature mortality due to NCDs, for people under 60 years of age, by at least 25% by 2020 [10]. To prevent NCDs, their modifiable risk factors, such as poor diet, insufficient physical activity, smoking and excessive alcohol consumption [11–14], must be reduced. These risk factor distributions at the population-level can potentially be changed by promoting lifestyle changes and enabling the environments where people live or work through policy and programs. Population-level interventions refer to policies or programs that aim to mitigate the distribution of health risk by addressing the underlying socio-economic, environmental, behavioral or cultural conditions in which people live and work [13]. There is a growing body of evidence that highlights both health and economic gains of interventions at population-level and that encourages low consumption of tobacco, alcohol, and salt; improved awareness of healthy lifestyle; increased excise taxes; and enhanced regulations [8, 13, 14].

**Fig. 1:**
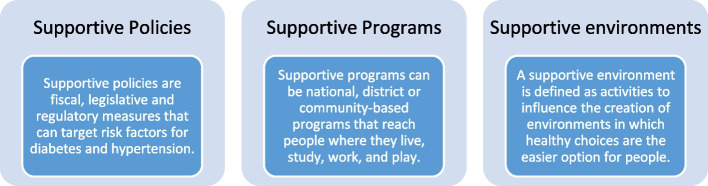
WHO DPAS Strategy

South Africa has several comprehensive programs that target NCD risk factors and most of these programs focus on unhealthy diet, tobacco use, alcohol consumption, and physical inactivity [15]. It is important to prioritise the examination of the policymaking process and subsequent programmes to understand context-specific issues that may be raised [16]. This qualitative study forms part of a larger study, a “Situational analysis on population-level interventions targeting risk factors for diabetes and hypertension in South Africa” [15]. Similarly to the larger study, we used the WHO Global Strategy for Diet, Physical Activity, and Health (WHO DPAS) [17] as a framework, and focused on three large categories to explore population-level interventions: supportive policies, programs, and environments (Fig. 1).

The purpose of this qualitative study was to explore expert decision makers perceptions and experiences of planning for population-level interventions, targeting risk factors for diabetes and hypertension, in South Africa.

## Methods

This study adopted a qualitative approach and used interviews and a focus group to collect data. These interviews took place in April - June 2021 using Microsoft Teams or Zoom due to the Coronavirus Disease (COVID-19) social distancing restrictions. Ethics approval was provided by the Health Research Ethics Committee at Stellenbosch University (N19/01/001). Participants completed a written consent form, which was required to be signed and returned to us before the interview could be scheduled. Verbal consent was also acquired at the start of the interviews. The consolidated criteria for reporting qualitative research [18], a 32-item checklist for research using interviews and focus groups, was used as a reporting guideline for this manuscript (Additional File 1).

### Participants and sampling

We aimed to interview expert decision makers that were considered as well informed and had participated in the planning and developing of policies, programs or supportive environments for prevention and management of diabetes and hypertension in South Africa. As this study was part of a larger study [19] involving a document review, we used the document review to inform the mapping of stakeholders to be involved in the key informant interviews by identifying experts in supportive policies, programs and enabling environments for NCD prevention in South Africa. Using purposive sampling, we reached out to stakeholders on the list and additionally requested referral information for other stakeholders meeting the inclusion criteria. We invited 28 managers, directors, and policy makers, with more than 5 years of working experience within the NCD policy and research environment to participate in individual interviews or the focus group, of which 15 participants agreed. Due to the overburdening of the health system, lockdown measures, and reprioritization of government and industry resources during the COVID-19 pandemic some participants did not have the time and capacity to participate in the study.

Participants were representative of various sectors (Table 1), at national and provincial government departments, and agencies involved in NCD related programs (i.e. Departments of Health, Department of Agriculture and Land Reform, Department of Human Settlements, the National Drug Authority of South Africa) as well as representatives of NGOs, the food industry (National Cancer Association of South Africa and Unilever Ltd); and representatives of academic and research institutions (South Africa Medical Research Council and Stellenbosch University).

**Table 1:** Sectors represented by participants

Participant	Sector
1,5,9,10, 13,14	National Department of Health
2	Central Drug Authority
3	Provincial Government - Health
4	Food Industry
6	National Department of Human Settlements
7	Academic
8	South African Medical Research Council
11, 15	Department of Agriculture
12	Cancer Association of South Africa (CANSA)

### Data collection and tools

A semi-structured interview guide (Additional File 2) was designed within this study, prior the conduct of the interviews. We conducted 12 individual interviews and 1 focus group (with 3 participants). Interviews were conducted in English, by LH and JUN over Microsoft Teams or ZOOM (due to the COVID-19 restrictions) and lasted between 45 min and 2 h. Interviews were audio recorded and were transcribed verbatim, then coded using NVivo [20]. All transcripts were audited for accuracy against the audio and JUN, who conducted the interviews or facilitated the focus group discussion, confirmed that the text was assigned to the correct speakers in the focus group. Four transcripts were returned to the transcribers to be audited against the audio files for a second time.

### Data analysis

The qualitative data was analysed thematically, independently at first, and then in duplicate by LH and JUN, using the WHO DPSA [17]. After independent analysis, LH and JUN discussed all codes and themes until consensus was reached and the analysis was completed.

The five stages for a qualitative thematic analysis as described by Pope and Mays [21] was applied. These steps include familiarisation with the data, identifying a thematic framework, indexing through applying the thematic framework by coding, synthesising data into categories called charting, and mapping and interpretation. An analytical grid of key themes was developed in line with the study aims and familiarisation with the first few transcripts, and then applied to the rest of the transcripts. A constant comparison process was used during analysis. Relevant data was identified, examined, and compared with other data to extract codes and determine dominant themes.

We discussed data saturation and agreed that for policy and programs enablers and challenges we had reached data saturation. However, data saturation was not reached under the area of supportive environments. Although there was more to explore, the COVID-19 pandemic posed serious challenges to the availability of stakeholders, and we therefore concluded the study.

### Reflexivity

This study was designed, piloted, implemented, and reported by three authors (LH, JUN, and TY). We, the authors all identify as female and from the African region. We have combined expertise in qualitative, quantitative, and mixed method research with backgrounds in psychology, public health, global health, social justice, evidence-informed policies, epidemiology, and biostatistics. Our multidisciplinary team was able to analyze and discuss findings from varying perspectives and stances. Through our networks and associations, we had access to policy makers and potential participants-some of which we knew and were familiar with our work. This helped us to further build the existing rapport we had with participants before, during and after the interviews. We kept detailed notes of the interviews, team meetings, and data analysis decisions to support the transparency of the research process. The findings of the study were shared and discussed with participants online in the follow up stakeholder workshops.

## Results

Through discussion and analysis of the data we interpreted the participations perceptions of population-level interventions, namely, policies, programs and enabling environments, and the enablers and challenges.

### Enablers

Key enablers for policy formulation included, stakeholders’ engagement and collaboration, contextualization of policies and programs, and evaluation and organic growth. A summary of the themes that emerged related to enablers for formulation or development of supportive policies, programs and enabling environments can be viewed in Table 2 and illustrative quotes in Additional File 3.

**Table 2:** Enablers of formulation of supportive policies, programs and enabling environments

Broad theme	Supportive policies	Supportive programs	Enabling environments
**Stakeholders’ engagement and collaboration**	Using strategic framework to inform policy formulation. Community engagement. Transversal and interdepartmental consultation. Research partnerships.	Partnering with universities and other experts.	Partnerships to enable school environments.
**Contextualisation of policies and programs**	Contextualizing policies. Using a comprehensive public health approach.	Being responsive to the needs of the community.	Free and fun programs for homes and communities. Community training and guidelines. Balancing the economic and nutritional needs of people. Reduction of sugar in products and restaurants having more healthy options. Encourage green and open spaces.
**Evaluation and organic growth**	Having a monitoring and evaluation plan to capture change, consumer perspectives and other information to inform future policies formulation.	Integrating ongoing quality improvement. Planning for use of multiple methods for evaluation. Incentivised training of community evaluators. Organic growth from smaller initiatives.	

#### Stakeholders’ engagement and collaboration

Community engagement combined with transversal (including industry and academia) and interdepartmental consultation, as well as partnerships, were mentioned by most participants. On the provincial level, ‘*transversal input’* was valued as *“a collaborative process being driven by the policy unit*” (Participant 3). When reflecting on the Salt Reduction Policy [22], for example, Participant 4 said, *“I know that there was a consultative process…there was quite a process of engagement with the food industry so that the industry could play its part in registering the amounts of salt in the products that contribute the most salt in South African diets.”* Participants reported that contextualised and adaptive policies are important when referring to the existing evidence on effective policies internationally, and research partnerships were important to inform new policies and for the conduct of new research. In describing the planning for the Western Cape on Wellness! (WOW!) program, Participant 3 related: *“I don’t have all the expertise, I then locked in University of Cape Town, University of the Western Cape, Stellenbosch University and now Cape Peninsula University of Technology in a contract that is called the TIREC – The Training, Implementation, Research and Evaluation committee [that show] that we all the experts in terms of physical activity, healthy eating, counselling etc. together”*. WOW! is a healthy lifestyles-promoting partnership programme of the Western Cape Government. The overarching aim of WOW! is to promote health, reduce health inequalities and strengthen social inclusiveness/connectedness by co-creating enabling environments for sustained healthy lifestyle choices throughout our life course. Partnering with universities and other experts was an important enabler for the WOW! program. Partnerships with schools to increase physical activity were found to be important for planning for enabling environments. Participants of the WOW! programme shared that the creation of enabling environments was challenging and influenced by both community and government level factors. An important enabler of creating supportive enabling environments was partnerships and using a community risk assessment tool that “*assesses their assets and risks in their own environment for walking more”* (Participant 3).

#### Contextualisation of policies and programs

Contextualizing policies, being responsive to the needs of the community, and using a public health approach were key themes identified as enablers of supportive policy and program development. The Western Cape on Wellness (WOW!) initiative, for example, came about as a response to the needs of communities and across different governmental departments. Participant 3, on being responsive to community needs, reflected that they wanted to work *‘with the public…and across departments’* and through working with communities they had a better understanding about what the needs were and could *‘be as responsive as possible’.* Contextualization of programs included providing community training and guidelines for the WOW! programme: “*We had to come up with community guidelines to say that if someone wants to sponsor food, fantastic, but give them these guidelines*” (Participant 3). Guidelines were seen as useful for promoting enabling environments. *“The objective of creating and enabling environment for people to make healthy food choices and quite a few components of that that have been implemented*” (Participant 10). Further to these, participants perceived that striking a balance between the economic and nutritional needs of people was essential to planning for programs and enabling environments. *“People are producing food now, not necessarily for them just to consume it at household level, what we see here in South Africa is that most households, they want to produce in order for them to sell and be able to get an income from it” (Participant 15).* Participant 12 noted that to support enabling environments, “*It is important for government to also encourage green and open spaces*”.

#### Evaluation and organic growth

Participants highlighted the need for monitoring and evaluation of policy and programs to capture for example positive change, information about consumer perspectives and further information to inform future policy formulation. They mentioned setting targets and objectives that are specific, measurable, attainable, relevant, and time based (S.M.A.R.T.). Participant 14 said, *“Ideally, we need to develop an M&E framework for the strategy which speaks to the data collection and to ensure that the targets that we set in the strategy are S.M.A.R.T., the objectives are S.M.A.R.T., and the targets are measurable*”. For supportive programs, participants mentioned the need for program evaluation through working closely with communities using an *“ongoing improvement model”* (Participant 3) which requires partnerships and regular communication with co-creation. Incentivising community implementers and participants through an “*annual award ceremony based on the M&E data*” was reported by Participant 3 to promote program evaluation. Multi method evaluation strategies were perceived as enabling. There is an awareness amongst participants that partnerships with academic institutions can strengthen the research and evaluation of programs. Additionally, planning programmes that could organically grow from smaller initiatives showing positive results in evaluation was seen as an enabler. Participant 12 reported that the “*eKick Butt program started off as a support group”* which organically grew into the program it is today. The Cansa Association of South Africa's (CANSA’s) eKick Butt program is an online smoking cessation program. Through a series of emails, surveys and downloads, guidance and mentorship are provided to quit smoking and non-smoking becomes a lifelong habit.

### Challenges

Key challenges for supportive policy and program formulation, and to enable supportive environments included the lack of time and resources, lack of stakeholder consultation, regulations and competing priorities, and ineffective monitoring and evaluation. A summary of the themes that emerged related to enablers for formulation or development of supportive policies, programs and enabling environments can be viewed in Table 3 and illustrative quotes in Additional File 4.

**Table 3:** Enablers of formulation of supportive policies, programs and enabling environments

Broad theme	Supportive policies	Supportive programs	Enabling environments
**Lack of time and resources**	Lack of time and resources. Lack of funding for research to inform policy.	Lack of community resources.	
**Lack of consultation and stakeholder engagement**	Non-reliance on the bottom-up approach	Lack of consultation and stakeholder buy in.	Lack of consideration for community spaces.
**Regulations and competing priorities**	Competing interests between government and public sectors.	Unethical sponsorship and poor catering.	Informal vendors selling unhealthy foods. Unregulated advertising of unhealthy foods.
**Ineffective monitoring and evaluation**	Lack of electronic monitoring systems.	Lack of longitudinal or baseline data.	

#### Lack of time and resources

Participant 2 reported that lack of time resulted in the National Strategic Plan for the Prevention and Control of Non-Communicable Diseases 2020–2025 (NCD Strategy) [23] being developed without much stakeholder consultation: “*…it was quite a rushed process unfortunately there was not much time that was given to developing that initial NCD strategy, hence there was not much consultation that took place in developing that NCD strategy”.* Participants working in the research industry was challenged by the lack of funding to do research relevant to the local African context and that is responsive to policy formulation needs. *“Unfortunately, the sort of research that we do regarding policy might not be always the research that the funders are looking to fund. This is one of the big issues, that we’re get the right funding for the research that we need to achieve the policy we think is relevant for the country” (Participant 9).*

### Lack of consultation and stakeholder engagement

Lack of consultation and stakeholder buy-in had serious consequences on policy development as seen in the example of the National Policy on Food and Nutrition Security [24], “*when we got approval of that policy, there was a whole lot of criticism around how the department didn’t go through a thorough process of consultation with civil society”* (Participant 15). Without consultation and stakeholder buy-in, policies may be reverted to the beginning stages of policy formulation as with the Strategic Plan for the Prevention and Control of Non-Communicable Diseases 2013-17 [10]. Community buy-in was found to be a challenge when there was a non-reliance on the bottom-up approach as shared about the Salt Reduction policy [22]. “*The bottom-up approach is what is missing in terms of how policies are developed, so we end up not having enough civil society support with the policies that are supposed to benefit them”* (Participant 10). Additionally, limited engagement with research institutions and communities hindered the contextualisation of policies and programs to the geographical areas within communities with little to no safe, green, and open spaces. *“People will create their own play areas or spaces for them to gather within that community and you as an outsider may not be able to make any sense of just a structure of that settlement the layout of that settlement”* (Participant 6). Although identification and stakeholders’ engagement are part of the policy formulation process, it was acknowledged by most of the participants as a complex and time-consuming process. Hence, thorough consultation and engagements with key stakeholders particularly the civil society, and research institutions leaders do not always take place. Participants acknowledged that the process of engaging with communities sometimes was too slow.

#### Regulations and competing priorities

The competing interests between government and other sectors was found to have a detrimental effect on policies and programmes aimed at reducing the risk factors for hypertension and diabetes. A government participant related how lack of regulations led to industries choosing alternatives that hindered successful programming. *“If you negotiate and not regulate, people choose the easiest way out”* (Participant 10). Similarly, Participant 13 believed between government and the private sector that *“…in some instances with regard to regulation, there are times where we know we will never agree on certain things*” (Participant 13). Competing priorities also translated into unethical sponsorships and poor catering, with governments accepting sponsorship from McDonald’s and Coca Cola when planning programs. “*We passed a resolution at our last conference where we said that government should not be accepting money from those type of donors [unhealthy foods and beverages] and of course the Minister of Sports at that time said, well then how do we fund or how do we then allow programs to continue?”* (Participant 2). Echoed by Participant 10 who said, *“we are regulating Coke, the department of sports wants donations from Coke when they have these physical activities, so it is a bit challenging, and it sort of undermines everything”.* Participants reported that similar practices were found in communities with informal vendors being a *“huge problem, selling unhealthy food outside schools and schools selling very unhealthy food in the canteens”* (Participant 3). Economic security largely influenced people access to food and the unregulated advertising of unhealthy foods (Participant 3) made enabling the environment very challenging when considering new programmes-as this poses a competing priority to health lifestyles.

#### Ineffective monitoring and evaluation

Barriers to evaluation data informing policy formulation were the lack of longitudinal or baseline data, lack of electronic monitoring systems, and competing interests between government and public sectors. When putting a policy in place, the lack of data to measure the impact of population-level interventions posed a barrier to refining and improving policies as exemplified in the Strategic Plan for the Prevention and Control of Non-Communicable Diseases 2013-17 [10] and the Regulations Relating to the Reduction of Sodium in Certain Foodstuffs and Related Matters [25]. Participant 14 perceived the lack of electronic mechanisms and infrastructure a limitation saying, “*We don’t have electronic mechanisms in place or the infrastructure in place for the targets that have been set and connecting data manually becomes a little bit stressful, because we cannot collect quality data”.* Program evaluation was found to be compromised by the lack of resources and evaluation frameworks, health officers feeling overburdened, and a lack of computer literacy and internet accessibility. Participant 12 relayed how the lack of evaluation frameworks presented a challenge by saying, *“…on paper it is great, is it having an impact? I do not know….”* Neglecting to do sufficient monitoring and evaluation compromised the effective planning for future policies and programs.

## Discussion

The South African government aims to reduce premature mortality (under 60 years of age) from NCD Strategy [23]. In order to do this, the combination of policies, programs and supportive environments should be, and are, targeted to reduce the risk factors for hypertension and diabetes, in line with the WHO best buys [14]. The findings of this qualitative study show the enablers and challenges for the planning of targeted population-level interventions. Partnerships, multisectoral approaches, community engagement and empowerment, responsive contextualized policies developed using a strategic framework, supportive enabling environments, and ongoing monitoring and evaluation to inform policy, and programme planning were common enablers. Conversely, the challenges found were lack of time and resources, lack of consultation and stakeholder consultation, regulations and competing priorities, and ineffective monitoring and evaluation systems.

The SA National Policy Development Framework (NPDF) [26], sets clear principles for effective policy development, emphasizing that it must be contextualized and responsive to people needs, and the public must be encouraged to participate. When the correct procedure for public comment and input is not followed in the planning phase, the policies not only run the risk of not being delivered in a contextually relevant way but may also be rejected by the public [23, 27, 28]. Non-reliance on the bottom-up approach can have serious consequences on the time taken to move from policy formulation to implementation [27]. Naude and colleagues [28] found that policy making processes are often lengthy and complex, and often include back-and-forth consultations with many diverse stakeholder groups. Most participants expressed some challenges related to formulation of supportive polices, there was a consensus that there is a room of improvement for formulation of certain supportive policies where thorough engagement with stakeholders could take place [29]. These policies include the national strategic plan for prevention of NCDs (2020–2025), the policy on salt reduction, excises taxes on sugar and sweeten beverages as well as smoking. Although, there is no definitive list of what sectors should be involved in collaborative planning, the core partnerships include those across government, civil society, and community members, as well as between different levels and departments in government [30, 31].

Formulations of policies and the planning of programs are largely reliant on monitoring and evaluation data as prescribed in the National Policy Development Framework [26]. This study found that most evaluation data was unreliable and did not support the strategic development of policies. Lack of baseline data and not using evidence for policy making creates opportunities for the competing priorities of stakeholders [32], which can have detrimental effects on the formulation of future policies and programs [33, 34]. This can lead to unethical sponsorship and unregulated advertising of unhealthy foods [35]. The socio-economic status of low-income communities create ideal targets for large corporations to sponsor programs with unhealthy food and beverages, when other sponsorships are not as available – and government departments become reliant on these corporations when planning. There is a wealth of public health research which indicates that food promotion has a direct effect on peoples behaviours related to food and beverages [36]. The main drivers of planning for policies and programs were the current contextual realities, costs, logistics, and people (clinicians, NGOs, funders) [28]. Collaborative planning and engagement [30], the re-evaluation of the aims and priorities of policies and programs, and reliable evaluation data are essential in order to successfully plan and formulate policies and programmes to reduce the risk factors for hypertension and diabetes at a population-level.

### Strengths and limitations

This study was conducted by trained qualitative researchers familiar with policy analysis. Coding and analysis were conducted independently and induplicate. This study presents the experiences and perceptions of policy makers and civil society representatives regarding planning and developing population-level interventions targeting risk factors of diabetes and hypertension in South Africa. Due to the COVID-19 pandemic we were unable to conduct the interviews face to face and therefore our experiences of the interviews may have missed non-verbal cues. Participants were willing to engage, and we were concerned that they may be protective of their relevant departments or places of work. Participants felt comfortable speaking freely after signing the consent forms and we continued to reassure participants of their rights within the study, throughout the data collection. Additionally, we used member checking to verify with participants that they agreed with the results and synthesis of their data. We determined that saturation on enabling environments was not reached, as the time to explore the relevant data was minimal as compared to policies and programs, due to the overwhelming demands of these structures on COVID-19 responses and access to reliable connectivity, especially for NGOs. However, adopting ethical research practices, as well as using COREQ reporting guideline, we present the study transparently.

## Conclusion

Findings from this study contribute further understanding of the enablers and barriers of policy and program planning that addresses the risk factors for hypertension and diabetes in SA. Policies and programs must account for the needs of the public and the historical and socio-economic climate, with patient representation at both the planning and implementation phase. Feasibility and sustainability of programs can only be ensured when the resources are provided, and environments enabled to promote behavior change on a population-level. There is a limited amount of monitoring and evaluation data due to lack of funding. Setting up evaluation systems, implementation research and policy analysis is needed. Assessment of the needs of communities to effect change must be carefully and sensitively executed, by partnering with experts such as academic institutions and civil society. Overall, planning for policy and programs should prioritize the needs of communities, as well as the social and environmental factors. Support for these activities should come from governmental leaders and managers, along with their continued commitment to enhance successful implementation. A holistic public health approach, which is contextually relevant, and evidence informed, is considered best practice in the formulation of population-level interventions.

## Supplementary Information

**Additional file 1.** Reporting Guideline.

**Additional file 2.** Interview guide for stakeholders.

**Additional file 3.** Illustrative quotes of enablers.

**Additional file 4.** Illustrative quotes of challenges.

## Data Availability

All data generated or analysed during this study are included in this published article [and its supplementary information files] and further information is available from the corresponding author on reasonable request.

## Abbreviations

CANSA: The Cancer Association of South Africa
COVID-19: Coronavirus Disease
CVD: Cardiovascular disease
DPAS: Diet, physical activity, and health
KII: Key informant interview
LMIC: Low- and middle-income countries
M&E: Monitoring and evaluation
NCD: Non-communicable diseases
NCD Strategy: National Strategic Plan for the Prevention and Control of Non-Communicable Diseases 2020–202
NGO: Non-governmental organization
NPDF: National Policy Development Framework
S.M.A.R.T.: Specific, Measurable, Attainable, Relevant, and Time based (S.M.A.R.T.)
SSA: Sub-Saharan Africa
SA: South Africa
WHO: World Health Organization
WOW!: Western Cape on Wellness!
