# Supplementary material for: Decision makers perceptions and experiences of developing population-level interventions targeting risk factors for hypertension and diabetes in South Africa: a qualitative study
Source: BMC Health Serv Res. 2023 Feb 11;23:146. doi: 10.1186/s12913-023-09135-x (PMC9918811; doi:10.1186/s12913-023-09135-x)
Supplement: Supplementary file 1 — Additional file 1. Reporting Guideline. [file 12913_2023_9135_MOESM1_ESM.docx]

*Additional File 1. Reporting Guideline*

| **No. Item** | **Guide questions/description** | **Reported on Page #** |
| --- | --- | --- |
| **Domain 1: Research team and reﬂexivity** | | |
| 1. Interviewer/facilitator | Which author/s conducted the  interview? | 6 |
| 2. Credentials | What were the researcher’s credentials? | 19 |
| 3. Occupation | What was their occupation at the time of  the study? | 19 |
| 4. Gender | Was the researcher male or female? | 7 |
| 5. Experience and training | What experience or training did the  researcher have? | 7 |
| 6. Relationship with participants established | Was a relationship established prior to study commencement? | 7 |
| 7. Participant knowledge  of the interviewer | What did the participants know about  the researcher? | 8 |
| 8. Interviewer  characteristics | What characteristics were reported  about the interviewer/facilitator? | 7 |
| **Domain 2: study design** | | |
| 9. Methodological  orientation and Theory | What methodological orientation was  stated to underpin the study? | 4 |
| 10. Sampling | How were participants selected? | 5 |
| 11. Method of approach | How were participants approached? | 5 |
| 12. Sample size | How many participants were in the  study? | 6 |
| 13. Non-­‐participation | How many people refused to participate?  or dropped out? Reasons? | 5 |
| 14. Setting of data collection | Where was the data collected? | 5-6 |
| 15. Presence of non-­‐  participants | Was anyone else present besides the  participants and researchers? | 6 |
| 16. Description of sample | What are the important characteristics  of the sample? | 6 |
| 17. Interview guide | Were questions, prompts, guides provided by the authors? | 6 and Additional File 2 |
| 18. Repeat interviews | Were repeat interviews carried out? | 6 |
| 19. Audio/visual recording | Did the research use audio or visual recording to collect the data? | 6 |
| 20. Field notes | Were ﬁeld notes made during and/or | 6-8 |

|  | after the interview? |  |
| --- | --- | --- |
| 21. Duration | What was the duration of the interviews | 6 |
| 22. Data saturation | Was data saturation discussed? | 7 |
| 23. Transcripts returned | Were transcripts returned to  participants for comment and/or correction? | 7 |
| **Domain 3: analysis and ﬁndings** | | |
| 24. Number of data coders | How many data coders coded the data? | 7 |
| 25. Description of the  coding tree | Did authors provide a description of the  coding tree? | Additional File 3-4 |
| 26. Derivation of themes | Were themes identiﬁed in advance or  derived from the data? | 7 |
| 27. Software | What software, if applicable, was used to manage the data? | 7 |
| 28. Participant checking | Did participants provide feedback on  the ﬁndings? | 7 |
| 29. Quotations presented | Were participant quotations presented to illustrate the themes/ﬁndings? Was  each quotation identiﬁed? | Additional File 3-4 |
| 30. Data and ﬁndings  consistent | Was there consistency between the data  presented and the ﬁndings? | 8-17 |
| 31. Clarity of major themes | Were major themes clearly presented in the ﬁndings? | Table 2-3  Additional File 3-4 |
| 32. Clarity of minor  themes | Is there a description of diverse cases or  discussion of minor themes? | 8-17 |
