# Supplementary material for: Decision makers perceptions and experiences of developing population-level interventions targeting risk factors for hypertension and diabetes in South Africa: a qualitative study
Source: BMC Health Serv Res. 2023 Feb 11;23:146. doi: 10.1186/s12913-023-09135-x (PMC9918811; doi:10.1186/s12913-023-09135-x)
Supplement: Supplementary file 2 — Additional file 2. Interview guide for stakeholders. [file 12913_2023_9135_MOESM2_ESM.docx]

**Additional File 2: INTERVIEW GUIDE FOR STAKEHOLDERS**

**Situational analysis of population level interventions targeting risk factors for diabetes and hypertension in South Africa.**

**INTERVIEW GUIDE**

Introduction

Good (morning/afternoon/evening), My name is _______________________________; today I will be conducting a situational analysis to identify interventions for prevention of diabetes and hypertension in South Africa.

This situational analysis aims to provide policymakers with the best available evidence on which to base policy and practice, by identifying and describing the types of current population level interventions being implemented in South Africa. The objective of this interview is to describe the most relevant interventions, including their planning, implementation, and evaluation.

For this study the risk factors we are focusing on include tackling tobacco use, harmful alcohol use, unhealthy diets, and physical inactivity.

In particular, we are interested in Population-level interventions which are policies or programs that aims to mitigate the distribution of health risk by addressing the underlying socioeconomic, environmental, behavioural, or cultural conditions in which people live and work. Population - level interventions of interest are those population-level interventions that are implemented at the level of governmental or political jurisdictions only (e.g., cities, regions, countries). These types of interventions can, in principle, be delivered by government bodies or non-governmental organizations.

To obtain reliable information we request that you answer the questions that follow as frankly as possible. Your views are important in this research. There is no right or wrong answer. It is your knowledge and opinion that count. The information you give to us will be kept confidential. You will not be identified by name or address in any of the reports we plan to write.

The interview will take not more than 60 minutes (PLEASE REQUEST INFORMED CONSENT)

Just to confirm that I have your details right: (USE THE SEPARATE FORM TO COMPLETE THE INFORMATION BELOW)

**PARTICIPANT DEMOGRAPGHICS**

| **Question** | **Response** |
| --- | --- |
| Name and Surname |  |
| Gender |  |
| Organisation Name |  |
| Contact Details (phone & email) |  |
| Position |  |
| Role/Responsability (ies) |  |
| Year started working at this organisation |  |
| Year started working in this position/role |  |

REQUEST TO TURN ON RECORDERS AT THIS POINT

Please note: Each key informant does not have to answer all the questions. Stakeholders will be assigned on which section to fill based on their background and experience.

**SECTION I: INTRODUCTION**

1. To what extent are you involved in the prevention of diabetes/hypertension? [direct or indirect: e.g., managing program to encourage healthier diet]

**Diabetes Hypertension**

1. To what extent is diabetes prevention a health priority in South Africa? [Probe to provide explanation of the answer]

**OR (proceed to questions 3 if person is involved in both diabetes and hypertension programs)**

1. To what extent is hypertension prevention a health priority in South Africa? Should it be of a higher priority? **[Probe to provide explanation of the answer]**

**Interview Notes:**

**Key persons referred too: ________________________________________________________________________________________________________________________________________________________________________________________________________________________________________________________________________________________________________________________________________________________________________**

**Key documents referred too:**

**__________________________________________________________________________________________________________________________________________________________________________________________________________________________________________________________________________________________________________________________________________________________________________________________________________________________________________________________________**

**SECTION II: SUPPORTIVE POLICIES**

***Definition Supportive Policies:*** *are fiscal, legislative, and regulatory measures that can target risk factors for diabetes and hypertension? (e.g., Introducing a policy on replacement of trans-fats with polyunsaturated fats)*

We would like to know more about national or provincial policies/legislation/strategies or guidelines that target the risk factors for diabetes and hypertension that are currently used in South Africa. We have provided a checklist that we have put together from our desk review of policies that target risk factors for hypertension and diabetes.

| **Risk factors** | **Policies** | **Year of inception** | **Tick response** |
| --- | --- | --- | --- |
| *Tobacco use -Smoking* | Tobacco Products Control Act 21 | 1993 |  |
|  | Tobacco Products Control Regulations | 1994 |  |
|  | Tobacco Products Control Amendment Act 23 | 1999 |  |
|  | Tobacco Products Control Amendment | 2000 |  |
|  | Tobacco Products Control Amendment Act 25 | 2007 |  |
|  | Tobacco Products Control Amendment Act 28 | 2008 |  |
|  | Tobacco Products Control Amendment regulations | 2011 |  |
|  | Control of Tobacco products and electronic delivery systems Bill | 2018 |  |
| *Alcohol consumption* | Liquor Products Act 60 | 1989 |  |
|  | National Liquor Act 59 | 2003 |  |
|  | National Liquor Regulations | 2004 |  |
|  | Western Cape Liquor Act | 2008 |  |
|  | Gauteng Liquor Act | 2013 |  |
|  | City of Cape Town Liquor bylaw | 2014 |  |
|  | National Drug Masterplan (2013–2017) | 2013 |  |
| *Unhealthy diet/nutrition* | Regulations relating to trans-fat in foodstuffs | 2010 |  |
|  | Salt Reduction Regulations | 2013 |  |
|  | Taxation of Sugar Sweetened beverages (SSBs) Bill | 2016 |  |
|  | Roadmap for nutrition in South Africa (2012-2016) | 2012 |  |
|  | Food and Nutrition Security Policy | 2013 |  |
|  | Strategy for Prevention and Control of Obesity (2015-2020) | 2015 |  |
|  | Integrated Food security strategy | 2002 |  |
| *Physical inactivity* | Schools Act 84 | 1996 |  |
|  | National Sports and Recreation Act | 1998 |  |
|  | Promotion of Physical Activity in Older persons | 2011 |  |
|  | National Sports and Recreation strategic plan (2012-2016) | 2012 |  |
|  | National Strategic Plan for NCDs (2013-2017) | 2013 |  |

1. Are there any policies missing on this list?
   1. Probe for relevant information of missing policy (risk factor, reach, target population)
2. Please tick/select the ones that you were most involved with formulation of
   1. Description of the processes used for policy (ies) formulation. Probe:
   2. Which stakeholders were involved in the formulation of these policies? (Example: departments, parliamentarians, NGO, Civil society, manufacturers, industries, etc.)
   3. What do you see as gaps in the formulation of policies targeting risk factors for diabetes and hypertension?
3. Please tick/select the ones that you were most involved with the implementation of
   1. We would like to know more about the implementation of these policies. Probe:
   2. Who was involved in the implementation of these policies? (Who were the main drivers?)
   3. What do you see as facilitators for effective implementation of these policies?
   4. What do you see as barriers for effective implementation of population level policies for prevention of diabetes and hypertension?
   5. What are the areas of improvement for effective implementation of policies targeting risk factors for diabetes and hypertension?

1. Which policies do you think have worked and why?
2. How were these policies monitored or evaluated?

**SECTION III: SUPPORTIVE PROGRAMS**

***Definition of Supportive Programs:***  *are programs can be national, district or community-based programs that reach people where they live, study, work, and play. (E.g., Media campaign for the benefits and showcases of exercising on TVs, or putting calorie points for food items, etc.)*

We would like to know more about national or provincial programmes or initiatives that target the risk factors for diabetes and hypertension that are currently being implemented in South Africa. We have provided a checklist that we have put together from our desk review of programmes that target risk factors for hypertension and diabetes

1. Could you mention other programmes or initiatives that you might be aware that are not listed on the table below?

| **Risk factors** | **Programmes** |
| --- | --- |
| ***Unhealthy diet/nutrition*** | *National School Nutrition Program* |
|  | *Integrated nutrition programme* |
|  | *Healthy Food -Discovery Health* |
|  | *Making the Difference through Nutrition* |
|  | Salt watch Campaign |
|  | Western Cape on Wellness (WOW!) |
| **Tobacco use** | National Quit line |
|  | CANSA's eKick Butt |
|  | Smokenders |
| **Physical inactivity** | Move for Health Day |
|  | National recreational day |
|  | Big walk Day |

| **Risk factors** | **Programmes or initiatives** | **Coverage (National/Provincial)** | **Targeted population** | **Evaluated (yes/no)** |
| --- | --- | --- | --- | --- |
| **Tobacco use (Smoking)** |  |  |  |  |
| **Unhealthy diet (e.g., Salt, Sugar, trans-fats, etc.)** |  |  |  |  |
| **Harmful use of Alcohol** |  |  |  |  |
| **Physical inactivity** |  |  |  |  |
| **Obesity** |  |  |  |  |

1. Please select the programmes you have been/are involved in.
2. Can you describe how these programmes mentioned above were developed and implemented? (Probe: What was the process?)

8.1. Which stakeholders were involved in the development and the implementation of these programmes? (Example: departments, parliamentarians, NGO, Civil society, manufacturers, industries, etc.)

8.2. What do you see as gaps in the development and the implementation of these programmes targeting risk factors for diabetes and hypertension?

8.3. What do you see as barriers for effective implementation of these programmes?

8.4. What do you see as facilitators for effective implementation of these programmes?

8.5. What are the areas of improvement for effective implementation of these programmes?

1. Which programmes do you think have worked and why?

1. Which programmes have been evaluated? Can you tell me more? (Probe: Impact or process evaluations)

**SECTION IV: SUPPORTIVE ENVIRONMENT**

***Definition of Supportive Environment:***  *defined as activities to influence the creation of environments in which healthy choices are made easier for people. (e.g., building sports, recreational facilities as well as safe spaces for children to play, access to healthy, etc.)*

1. We would like to know more about *initiatives/activities that influence the creation of environments in which healthy choices are made easier for people* that target the risk factors for diabetes and hypertension that are currently used in South Africa?
2. Could you please mention interventions/ initiatives you may be aware of?

| **Supportive environment interventions (***e.g., building sports, recreational facilities as well as safe spaces for children to play, access to healthy food in the community, limited access to alcohol)* | **Description of the Initiative** | **Coverage /Scale (National/Provincial)** | **Targeted population** | **Evaluated (yes/no)** |
| --- | --- | --- | --- | --- |
|  |  |  |  |  |
| **Tobacco use (Smoking)** |  |  |  |  |
| **Unhealthy diet (e.g., Salt, Sugar, trans-fats, etc.)** |  |  |  |  |
| **Harmful use of Alcohol** |  |  |  |  |
| **Physical inactivity** |  |  |  |  |
| **Obesity** |  |  |  |  |

1. Can you tell us more about the planning and the execution of these interventions?

- *Which stakeholders were involved? (Example: departments, parliamentarians, NGO, Civil society, manufacturers, industries, etc.)*
- *Can you tell us more about the acceptability of these interventions?*

1. What do you see as gaps in planning and execution of these interventions targeting risk factors for diabetes and hypertension?
2. What do you see as barriers /challenges for effective planning and execution these interventions?
3. What are the areas of improvement for effective implementation of these interventions?
4. What do you see as facilitators for effective implementation of these interventions?

1. Which supportive environment interventions do you think have worked and why? (Probe: acceptability and feasibility)

1. Which supportive environment interventions have been evaluated? (Probe: Impact or process evaluations)
2. How are these interventions/activities being monitored or evaluated?
