# Supplementary material for: Decision makers perceptions and experiences of developing population-level interventions targeting risk factors for hypertension and diabetes in South Africa: a qualitative study
Source: BMC Health Serv Res. 2023 Feb 11;23:146. doi: 10.1186/s12913-023-09135-x (PMC9918811; doi:10.1186/s12913-023-09135-x)
Supplement: Supplementary file 3 — Additional file 3. Illustrative quotes of enablers. [file 12913_2023_9135_MOESM3_ESM.docx]

**Additional File 3: Illustrative quotes of enablers**

| **Supportive policies** |
| --- |
| **Using an existing or developing a new strategic framework to inform policy formulation**  *“We never had a strategy prior the National NCD Strategic Plan 2012-2015 and it was realised that it was imperative that we have a strategy for NCDs”* (Participant 1).  **Community engagement**  *“South Africa I think is really great in terms of formulating these policies in a consultative manner and trying to really tick the boxes into terms of what the recommendations are even from the World Health Organization”* (Participant 12).  **Research partnerships with policymakers**  *“We approach it as a unit. We do research we think can impact the policy directly in collaboration with the head authorities, so we don’t do it in isolation, we make sure that we incorporate the thought of the DOH, the direction they’re taking into the research, so much so that if we were to achieve something at the end, that should be able to be put into policy, so I think that partnership is key”* (Participant 8).  **Transversal and interdepartmental consultation**  *“Department of Health is doing a lot more to be consultative and inclusive in formulating policies. There are often times academic advisors are included and work closely with the Department of Health which I think is important. I think that signiﬁcant eﬀort has been made to get comment from other role players including industry…NGOs…and people that have the technical know-how to enable policies. I do think that that process has been good in terms of formulating policies”* (Participant 12).  **Contextualising policies**  *“There are guidelines when developing alcohol policy consumption most of the guidelines developed from other countries and from international organizations like the WHO…so we are informed by most of evidence based… and then we tried to adapt to suit our own country”* (Participant 9).  **Using a comprehensive public health approach**  *“Looking at it from health and medical perspectives we are saying what is best to follow a comprehensive policy public health approach”* (Participant 9).  **Monitoring and evaluation to capture change**  *“One of the positives that was identiﬁed by the report was that people started knowing or started recognizing non communicable diseases. Because before then non communicable diseases was like not even…that recognition, I don’t think currently it’s given the recognition that it deserves, it is still lacking as far as recognition is concerned but at least that strategy made people aware about the NCDs”* (Participant 14).  **Information about consumer perspectives**  *“The regulations have come into place and there was lots of work that went into making it happen, so consumer research from our side was to ﬁnd out how the product would be accepted and to ﬁnd out from a consumer point of view what changes we needed either in the product while still complying with the regulations. We also did research on what messaging we needed to go with the product to encourage behavior change”* (Participant 4). |
| **Supportive programs** |
| **Being responsive to the needs of the community**  *“…with the public, with the community…and through working with the communities and understanding more about what the needs are, and also working across diﬀerent departments, we tried to be as responsive as possible”* (Participant 3)*.*  **Partnering with universities and other experts**  *“I don’t have all the expertise, I then locked in UCT, UWC, Stellenbosch and now CPUT in a contract that is called the TIREC – The Training, Implementation, Research and Evaluation committee, contract, that we all the experts in terms of physical activity, healthy eating, counselling etc. together”* (Participant 3).  **Organic growth from smaller initiatives**  *“The eKick Butt program started oﬀ through a supportive group so it was people who were trying to quit smoking. The CANSA formulated a support group because it was realized that people needed support through the process and that is kind of how it began. And to my understanding this is a program where it has grown from being a supportive group to having regular kind of checking people who are trying to quit”* (Stakeholder 12).  **Using multiple methods for evaluation**  *“We do both qualitative and quantitative M&E all the time. We are currently in the process of doing a social network analysis with the champions and all our partners and that is done in partnership with our academic consortium that we have”* (Participant 3).  **Ongoing improvement model**  *“…we meet roughly quarterly, and come up with ideas, how we can address the problem, how do we make it exciting, how do we change it, and then we go back to the community and say okay let’s test this with you, see if this works, it’s very much a responsive creative, ongoing, organic model in a way.”* (Participant 3).  **Training and incentivising community evaluators**  *“On an annual basis we have what we call the WOW annual award ceremony, that now will be in its 5^th^ year. We look at the whole year’s M&E data, quantitative and qualitative, and we identify the top performing WOW groups and the champions who’ve really performed outstandingly. It’s about how innovative you are and despite all the odds, that you still achieve, and you make change. We can see each year it’s growing, when we started, we had about 80 to 100 guests, the past one we had in February this year, we had 320 guests”* (Participant 3). |
| **Enabling environments** |
| **Free and fun programmes for homes and communities**  *“In terms of physical activity, we started WOW! groups, you might have heard of, they oﬀer a structured program to promote physical activity, not only in the home environment but in the community, and also at work- so it’s diﬀerent populations or diﬀerent target groups…people meet, they walk, they network, and it’s just become quite a fun element”* (Participant 3).  **Partnerships to enable school environments**  (Participant 3) “WOW! has partnered with…  …v*arious companies including the Heart and Stroke Foundation” to “develop healthy tuckshop guidelines…”*  *…an undisclosed partner to test something new…it’s a PEER education model, or a social science program with the representative council of learners at schools are given the skills, the tools and an app to identify enablers and barriers to healthier lifestyles in a school environment…”*  *…Kubeka bicycles and gave 3000 bicycles to learners in the Paarl area to help them, not only to be more physically active, but also to live a healthy lifestyle and to improve their academic performance by not turning up late for school.”*  **Community training and guidelines**  *“We had to come up with an idea and we’re still working on it to give the community guidelines to say if someone wants to sponsor food, fantastic, but give them these guidelines and tell them anything on this list, that’s acceptable, so we’re trying to overcome that”* (Participant 3).  **Balancing the economic and nutritional needs of people**  *“People are producing food now, not necessarily for them just to consume it at household level, what we see here in South Africa is that most households, they want to produce for them to sell and be able to get an income from it. So now if there aren’t clear markets that has been achieved for these indigenous foods, people will not be so eager to produce them because there will not be any economic gains from it, it will not form part of the rural economist that we are talking about. People will be more eager to be involved in producing crops that are more likely to get them their income that they’re looking for” (Participant 15).*  **Reduction of sugar in products and restaurants having more healthy options**  *“The objective of creating and enabling environment for people to make healthy food choices and quite a few components of that that have been implemented…reduction of sugar in some products, the creating of new products which have less sugar or no sugar and restaurants coming up with new healthy options”* (Participant 10).  **Encourage green and open spaces**  *“It is important for government to also encourage green and open spaces”* (Participant 12). |
