# Supplementary material for: Decision makers perceptions and experiences of developing population-level interventions targeting risk factors for hypertension and diabetes in South Africa: a qualitative study
Source: BMC Health Serv Res. 2023 Feb 11;23:146. doi: 10.1186/s12913-023-09135-x (PMC9918811; doi:10.1186/s12913-023-09135-x)
Supplement: Supplementary file 4 — Additional file 4. Illustrative quotes of challenges. [file 12913_2023_9135_MOESM4_ESM.docx]

**Additional File 4: Illustrative quotes of challenges**

| **Supportive policies** |
| --- |
| **Lack of time and resources**  *“…it was quite a rushed process unfortunately there was not much time that was given to developing that initial NCD strategy, hence there was not much consultation that took place in developing that NCD strategy”* (Participant 2).  **Lack of funding for research to inform policy**  *“Unfortunately, the sort of research that we do regarding policy might not be always the research that the funders are looking to fund. This is one of the big issues, that we’re get the right funding for the research that we need to achieve the policy we think is relevant for the country”* (Participant 9).  **Non-reliance on the bottom-up approach**  *“The bottom-up approach is what is missing in terms of how policies are developed, so we end up not having enough civil society support with the policies that are supposed to beneﬁt them”* (Participant 10).  **Competing interests between government and public sectors** *“If you negotiate and not regulate, people choose the easiest way out. For instance, they will say to you that they will assist you in developing or creating gyms with funding. You know how much you must exercise after eating a bar of chocolate, so they want to do what they know doesn’t work and then at the end they’ll be saying government doesn’t want our support. So, we have been very challenged when it comes to working with industry”* (Participant 10).  *“And of course, all the private sector, in some instances with regard to regulation, I mean there are times where we know we will never agree on certain things”* (Participant 13).  **Lack of electronic monitoring systems**  *“We don’t have electronic mechanisms in place or the infrastructure in place for the targets that have been set and connecting data manually becomes a little bit stressful, because we cannot collect quality data”* (Participant 14). |
| **Supportive programs** |
| **Lack of community resources**  *“Another barrier we have is that communities, because of the lack of resources, they are so delighted if someone wants to give them food for a meeting, then they serve them the most horrible unhealthy food available”* (Participant 3).  **Lack of consultation and stakeholder buy in.**  *“…it’s the whole community engagement element that is not strong enough, that should take a stronger position in this because there’s still a tendency to do develop policy or even practice programs for people and not with the community”* (Participant 3).  **Unethical sponsorship and poor catering**  *“Also at community level, a big challenge, not only community, even government, is the poor catering, the disconnect between… association between lifestyles and diabetes, hypertension and what we eat, what we serve in hospitals, what we serve in canteens, total disconnect, which is really frustrating, another big barrier is sponsorship, ethical sponsorship and partnering doesn’t exist, we’re trying so hard with the WOW programme and yet still I will ﬁnd departments partnering with McDonalds and other unhealthy brands”*(Participant 3).  *“We passed a resolution at our last conference where we said that government should not be accepting money from those type of donors [unhealthy foods and beverages] and of course the Minister of Sports at that time said, ‘Well then how do we fund or how do we then allow programs to continue?’ Sports industries will have to come to an end. So, these are the issues. It all runs around finance, around money, how do they generate funding and money for themselves and what are the other opportunities that they can get to be able to sponsor and to run these programs”* (Participant 2).  *“I mean we are regulating Coke, the department of sports wants donations from Coke when they have these physical activities, so it is a bit challenging, and it sort of undermines everything”* (Participant 10).  **Lack of longitudinal or baseline data**  *“You know the main challenge that we had with the previous NCD strategy was that we didn’t have baselines, it was a bit of a challenge to measure because we didn’t know the premise we were moving from, that is one of the things that was raised by the report, the evaluation report says that…we were lacking as far as the setting of the base lines”* (Participant 5). |
| **Enabling environments** |
| **Informal vendors selling unhealthy foods**  *“Another big barrier we have in the community is the informal food vendors, huge problem, selling unhealthy food outside schools and schools selling very unhealthy food in the canteens, changing that is very hard, working with the education department is hugely challenging as I’m sure you all know, but it’s a long term process so it forces us to keep thinking creatively all the time, hence my argument earlier on, there was no point in us coming up with a policy about wellness, say implementing in schools, it can’t work, we are now 6 years later down the line, we’re still battling to get into schools and that’s not due to a lack of policy, it is just a lack of understanding, a lack of ﬁnding the hook to really work closely with them.”* (Participant 3)  **Lack of consideration for community spaces**  *“When we say to people walk more in your community…they say it’s unsafe”. (Participant 3)*  *“People will create their own play areas or spaces for them to gather within that community and you as an outsider may not be able to make any sense of just a structure of that settlement the layout of that settlement”. (Participant 6)*  **Unregulated advertising of unhealthy foods.**  *Another big challenge we have is advertising and again that’s something that national should look at, I know they are, but it’s a slow process, so we keep competing with the McDonalds, the Burger Kings, it’s very, very hard when you try to promote something and then the communities live in a food desert where there is nothing and that’s a massive challenge when we try to teach people, even cooking methods.* (Participant 3) |
